# Influence of PODE1 additive into ethanol-gasoline blends (E10) on fuel properties and phase stability

**DOI:** 10.1016/j.heliyon.2023.e22364

**Published:** 2023-11-17

**Authors:** Omar I. Awad, Bo Zhou, Zhenbin Chen, Kumaran Kadirgama, M.N. Mohammed, D. Ramasamy

**Affiliations:** a School of Mechanics and Electronics Engineering, Hainan University, Haikou, China; b Department of Petroleum Engineering, University of Kirkuk/College of Engineering, Kirkuk City, Iraq; c Mechanical Engineering Department, College of Engineering, Gulf University, 26489, Bahrain; d Department of Mechanics and Aerospace Engineering, Southern University of Science and Technology, China, Shenzhen; e Almaaqal University, College of Engineering, Department of Civil Engineering, Basra, 61003, Iraq; f Mechanical and Automotive Engineering Technology, University Malaysia Pahang, Pekan 26600, Pahang, Malaysia; g Advanced Nano Coolant-Lubricant (ANCL) Lab, Automotive Engineering Centre, Universiti Malaysia Pahang, Pekan 26600, Pahang, Malaysia

**Keywords:** Polyoxymethylene dimethyl ethers PODEn, PODE1, Gasoline-ethanol blend, Distillation curve, Boiling point

## Abstract

Polyoxymethylene dimethyl ethers (PODEn, n = 1–8) as an oxygenated fuel are a promising alternative fuel with a high oxygen concentration, a low C:H ratio, and no C–C bonds in their chemical structure. This could lead to smoke-free combustion. In this study, we chose to focus on PODE1 because of its lower cetane number, which makes it more suitable for use in spark ignition (SI) engines. However, its lower boiling point and octane number remain challenges. A low boiling point may lead to high vapour pressure and require storage and handling comparable to gaseous fuels. We investigated the effect of adding PODE1 to gasoline-ethanol blends (E10) on fuel properties, including distillation curve, octane number, phase stability, C/O/H ratio, heat of combustion, kinematic viscosity, and density. Our results showed that the blended fuels of E10 and PODE1 are stable up to 10 % PODE1, and there was no phase separation. Additionally, up to 10 % PODE1 additive had no significant side effect on the fuel properties of E10, particularly boiling point and octane number. Thus, work offers creative points by proposing a new candidate for additive fuel to gasoline-ethanol blends, which contributes to reducing the soot emission of GDI engines.

## Graphical abstract

**Figure:**
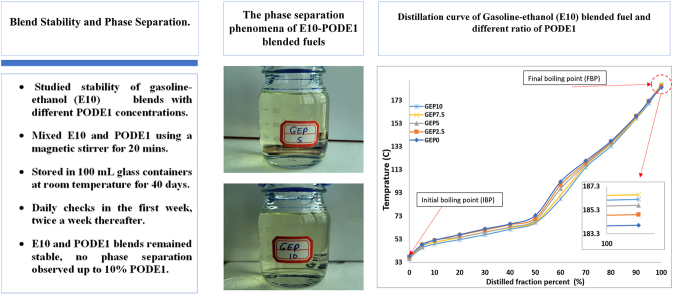

## 1 Introduction

These days, owing to higher emission requirements and the fast expansion of industry, there is a lot of focus on developing extremely efficient and clean internal combustion engines (ICEs) [1]. Gasoline is the main petroleum fraction separated from crude oil by distillation and it is used mainly as a fuel for ICEs [2,3]. One of the essential regulated emissions of ICEs is soot particulates, becoming a more serious issue as the regulation becomes stricter [4]. To reduce emissions and improve ICE performance, various new approaches have been developed, including improvements to the ICE injection and ignition systems, lubrication systems, thermal management control systems, and the use of alternative fuels.

Among these strategies, the integration of alternative fuelshas contributed greatly to the enhanced performance and reduced emissions of internal combustion engines (ICEs) [[5], [6], [7]]. Ethanol, sourced from renewable origins, shows promise as an effective option for spark-ignited (SI) engines due to its environmentally friendly characteristicsand high oxygen content [8]. Numerous investigations have explored the application of ethanol for ICE applications, with a particular emphasis on ethanol-gasoline blends developing as a viable solution for SI engines [9]. These blends offer has various benefits, including lowering HC and CO emissions, increasing combustion efficiency, and boosting octane number [10].

E10, a prevalent ethanol-to-gasoline blend consisting of 10 % ethanol and 90 % gasoline, has gained widespread usage, constituting over 98 % of gasoline in the United States to adhere to air quality regulations and augment octane ratings [11]. E10 has been gradually implemented in Europe since 2009 and was made essential across China in 2020 [12]. However, several studies have shown that as the ratio of ethanol increases, particulate matter emissions from GDI engines also increase. Despite this general belief, experimental results from laboratory-scale tests and engine tests have shown that the influence of ethanol on particulate matter and aromatic compounds is not clear-cut [13]. The emission of particulate matter is directly affected by fuel properties such as aromatic content, oxygen content, and volatility, and is dependent on the developments in vehicle technology [14]. In addition, it was found that there was a relationship with the molecular structure of fuel components, such as the proportion of oxygen content and aromatic hydrocarbons [15]. In other words, the physical and chemical properties of fuel, including the viscosity, boiling point, distillation curve [16], saturated vapour pressure, surface tension, molecular structure and components (carbon chain length, branching degree, saturation degree, double bond number and site, number of rings, C/H/O ratio) all play very important roles [17].

Polyoxymethylene dimethyl ethers (PODEn; n = 1–8) are considered promising alternative fuel candidates due to their low carbon-hydrogen (C: H) ratio, high oxygen content, and lack of carbon-carbon (C–C) bonds in their chemical structure, which may lead to smoke-free combustion [18]. PODEn molecules show a favorable mutual solubility with diesel. Previous studies have shown that up to 10 % of PODE_n_ blended with gasoline and diesel in compression ignition (CI) engines significantly impacts reducing soot. Awad, Ma [19] have been shown that the percentage of reduction among the soot, PM, and PN emissions increased by using PODEn (n = 1–8). Among the PODEn group, PODE1 reported a significantly lower soot emission than PODEn (n = 2–8) at the same blending ratio with diesel. Studies on the use of PODE1 as a diesel additive have indicated that it can produce almost soot-free diffusion flames without affecting NOx emissions [20,21].

However, key challenges to PODEn utilization as a gasoline additive or gasoline-ethanol blend in spark ignition engines are a lower boiling point, a lower octane number, and a higher cetane number. In high-temperature conditions, fuel pumps and fuel lines may boil if the fuel used in internal combustion engines (ICE) has a low boiling point [22]. The vapour leads to a reduction in the flow of fuel to the engine, which ultimately results in a loss of power, erratic engine performance, or the complete shutdown of the engine. Also, the lower boiling point cloud represents a challenge in fuel storage in pure form or blended with other fuels. The distillation curve, also referred to as the curve of boiling points, represents a fundamental and indispensable property of gasoline fuel within the context of its utilization and performance. This property holds critical importance due to its direct influence on the fuel's behavior during various stages of the combustion process and its compatibility with engines [22]. Gasoline is a complex combination of several hydrocarbons; therefore, it cannot be accurately described by a single boiling point but rather by a boiling range and some temperature data relating to the quantity of distilled fraction present. Furthermore, diesel (CI engine) types, such as the PODE3-7, prefer fuel with a higher cetane number than gasoline (SI engine).

The existing literature lacks comprehensive insights into the integration of PODE1 into Gasoline-Ethanol blends (E10) and its impact on fuel properties. The objective of this study is to bridge this gap by investigating the potential of PODE1-enhanced blends as a strategic solution to balance emissions reduction and engine, In this study, the focus is on PODE1 as a promising option for SI enginesdue to lower cetane number compared to PODEn (n = 2–8), which could be more suitable for SI engines; however, both the lower boiling point and octane number are still considered the main challenges. Pure PODE1 has a lower boiling point that may lead to high vapour pressure, necessitating storage and handling requirements comparable to those of gaseous fuel. Thus, the influence of PODE1 additives in gasoline-ethanol blends (E10) on fuel properties is mainly due to the distillation curve, octane number and phase stability has been investigated. In additional C/O/H ratio, heat of combustion, kinematic viscosity and density have been analysis and investigated. By addressing these challenges, we aim to shed light on the potential of PODE1-enhanced blends as a strategic means to balance emission reduction and engine performance in SI engines.

## 2 Experimental

### 2.1 Materials and methods

In this study, samples of ethanol and PODE1 were obtained from local commercial suppliers, and gasoline was purchased from a Sinopec petrol station in Shenzhen. The properties of pure gasoline, ethanol, and PODE1 are listed in Table 2. Blended samples were prepared with gasoline (90–81 %), ethanol (10–9%), and PODE1 (2.5–10 %) in the following proportions: GEP0 (100 % E10 + 0 % PODE1 by volume), GEP 2.5 (97.5 % E10 + 2.5 % PODE1 by volume), GEP 5, GEP 7.5, and GEP 10. The fuel properties of the blended fuels were measured using ASTM standards by SGS China and included the distillation curves, octane number, kinematic viscosity, density, heating value, boiling point, hydrogen, carbon, and oxygen content, as listed in Table 3. The research octane number (RON) was measured according to ASTM D2699-22, and the distillation range of fuel was determined according to ASTM D86-20b. The results of these analyses are provided in the supporting information.

## 3 Blend stability and phase separation

In this study, the mixing stability of gasoline-ethanol (E10) blended fuels with varying concentrations of PODE1 (0 %, 2.5 %, 5 %, 7.5 % and 10 %) was investigated to address the critical challenge of fuel instability [23]. The samples were prepared by mixing E10 and PODE1 using a magnetic stirrer for 20 min and stored in 100 mL glass containers at room temperature for 40 days to observe the long-term stability. The samples were checked daily during the first week and twice a week until the end of the 40th day period. The results showed that the blends of E10 and PODE1 were single-phase, indicating their stability and suitability for use as fuels, with no evidence of phase separation even at a concentration of PODE1 up to 10 %. These findings are displayed in Table 1 and are a significant outcome of the study.

**Table 1:** The phase separation phenomena of E10-PODE1 blended fuels.

Fuel blending ratio (%)			Elapsed time after mixing fuel (day)	
Gasoline	Ethanol	PODE1	**0**	**40**
90	10	0		
85.5	9.5	5		
81	9	10

**Table 2:** Physicochemical properties of Pure gasoline, ethanol and PODE1.

Properties	Unit	Test Method	Gasoline	Ethanol	PODE1
RON		ASTM D2699-22	95	103	–
Kinematic Viscosity 20C	mm^2^/s	ASTM D445-21e1	0.490	0.6675	0.360
Density at 20 C	g/cm^3^	ASTM D4052-22	0.769	0.790	0.865
Heat of combustion	MJ/Kg	M2521	43.50	21.2	22.44
Carbon (C)	% (m/m)	M1394	87.50	52.35	–
Hydrogen (H)	% (m/m)		12.50	13.25	–
Oxygen (O)	% (m/m)		0.00	34.4	42.1
Boiling point	°C	ASTM2887	27–225	75–80	42

**Table 3:** Physicochemical properties of blended fuel.

Properties	Unit	Test Method	GEP0 (E10)	GEP2.5	GEP5	GEP7.5	GEP10
RON		ASTM D2699-22	98	97.8	97.5	97.2	97
Kinematic Viscosity 20C	mm^2^/s	ASTM D445-21e1	0.6686	0.6675	0.6405	0.6004	0.6481
Density at 20 C	g/cm^3^	ASTM D4052-22	0.7452	0.7484	0.7509	0.754	0.7563
Heat of combustion	MJ/Kg	M2521	41.51	41.13	40.56	40.23	39.83
**C/O/H ratio**	(m/m)	M1394					
Carbon (C)			81.27	80.18	79.25	78.09	77.07
Hydrogen (H)			13.74	13.46	13.41	13.23	13.23
Oxygen (O)			5.6	6.36	7.34	8.63	9.7
**Distillation Temp**	°C	ASTMD86-20b					
Initial boiling point (IBP)			38.4	37.5	36	37.1	36.3
T50			73.5	70.9	69.2	68	67
T90			159.5	159	158.5	157.4	157.2
T95			172	172	172.7	172.3	170.3
Final boiling point (FBP)			184	184.9	185.7	186.6	186.2

## 4 Results and discussion

In this section, we present the outcomes of our analysis on the properties of pure gasoline, ethanol, and PODE1, both individually and in blended fuel compositions. Table 2, Table 3 provide a comprehensive overview of the properties of these substances.

### 4.1 Distillation analysis and fuel behavior

. We begin by discussing the distillation analysis of the fuel blends, which sheds light on their behavior in relation to temperature and volume distilled.Distillation curves, crucial for understanding fluid mixtures, reveal the boiling points of components in relation to volume [24]. The gasoline composition is made up of various hydrocarbons, each with a unique boiling point. The distillation curve is plotted at specific conditions, showing the increasing temperatures at which gasoline evaporates as the volume increases in increments of 5 %, 10 %, 20 %, 30 %, and so on [25]. The distillation curve is characterized by three key points, T10, T50, and T90, representing the temperatures at which 10 %, 50 %, and 90 % of the gasoline's initial volume has vaporized [26]. These temperatures signify the volatility of the fuel's light, medium, and heavy fractions, which in turn impact the engine's performance during different operating regimes [27]. The light fraction impacts the gasoline's flashpoint and the vehicle's starting ability, while the medium fraction affects engine performance due to its impact on density and viscosity. The heavy end has a significant impact on emissions and is an area of active research. In motor vehicles, the boiling point range of fuel affects properties such as density, viscosity, flashpoint, flow, and ignition, particularly at low temperatures. The boiling point curve determines the fuel's behaviour in the engine and its suitability. Low boiling points of ICE fuel can result in boiling pumps and pipes at high temperatures, which reduces fuel flow to the engine and can cause power loss, poor engine operation, or engine stoppage.

In summary, the distillation curve provides a graphical representation of the boiling temperature of a mixture plotted against the volume fraction distilled [28]. PODE1 has a low boiling point (42 °C) and high vapour pressure, which requires storage and handling requirements like gaseous fuels and could also impact the boiling point of E10 (GEP0).

**Fig. 1:**
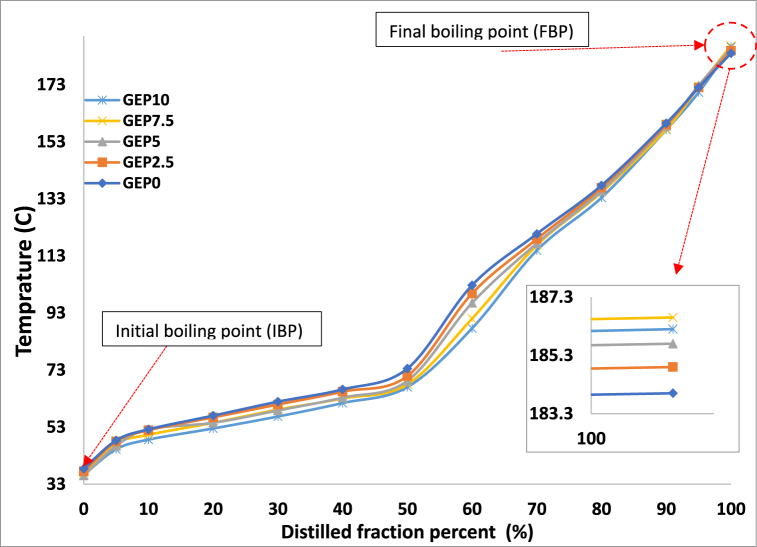
Distillation curve of Gasoline-ethanol (E10) blended fuel and different ratio of PODE1.

The results of the distillation analysis of E10 with varying concentrations of PODE1 are presented in Fig. 1. The distillation curves indicate that the PODE1 additives have little impact on the initial vaporization temperatures of the blends, but their effect becomes more noticeable towards the end of the distillation process, particularly at T50 and T60. The data reveals that the initial boiling point of the base E10 (GEP0) composition is slightly higher than that of the blends containing varying ratios of PODE1. However, the final boiling point of the blends decreases as the ratio of PODE1 increases. Despite the lower boiling point of PODE1, which could have an adverse impact on fuel performance, the combination with E10 may help to mitigate these effects. Additionally, the use of up to 10 % PODE1 additives can significantly reduce emissions, particularly soot emissions from gasoline direct ignition (GDI) engines.

### 4.2 Octane number

**Fig. 2:**
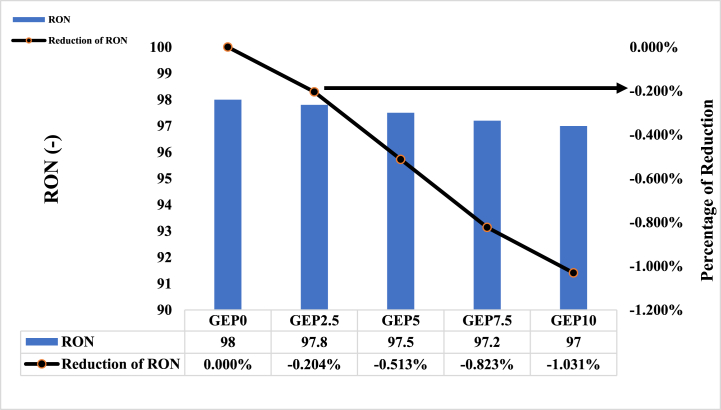
Research octane number of Gasoline-ethanol (E10) blended fuel and different ratio of PODE1.

The octane number is a metric used to assess the quality of gasoline. It is determined by measuring the gasoline's resistance to knocking, or its anti-knock properties, when burned in an engine [26]. If the fuel-air mixture ignites before the spark plug fires, it can lead to a reduction in engine efficiency and even damage to the cylinder heads. The octane rating of gasoline has a direct impact on engine performance since, as the compression ratio of the engine increases, the likelihood of pre-ignition also increases [27]. The use of gasoline with a higher-octane rating helps to prevent knocking and enhance engine efficiency. Fig. 2 shows the values and reduction percentage of the research octane number (RON) among gasoline-ethanol (E10) blends and different PODE1 additive ratios. It can be observed that the research octan number (RON) of the blends slightly decreased with an increase in the PODE1 ratio in the blends. This is attributed to the fact that PODE1 has lower RON and viscosity. The maximum reduction in the RON is 1.2 % at the highest ratio of PODE1 (GEP10).

### 4.3 Elemental composition

**Fig. 3:**
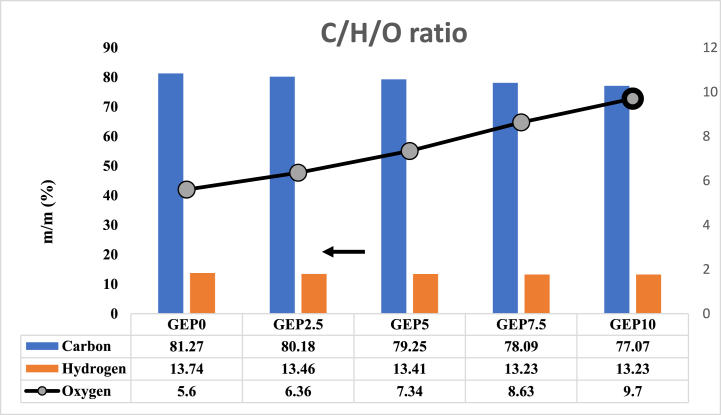
C/H/O ratio of Gasoline-ethanol (E10) blended fuel and different ratio of PODE1.

**Fig. 4:**
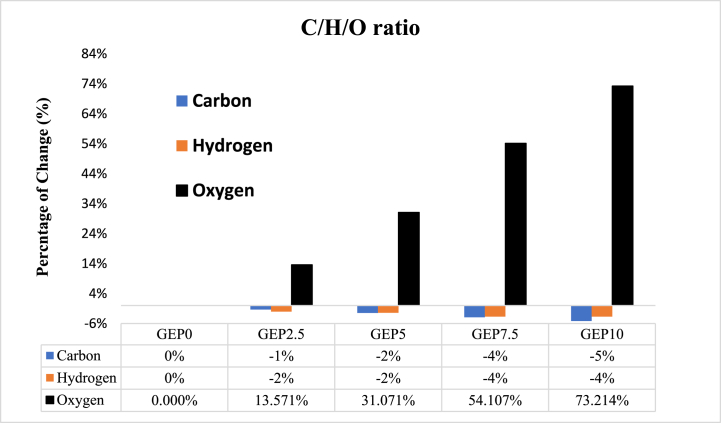
Percentage of reduction for C, H, and O of Gasoline-ethanol (E10) blended fuel and different ratio of PODE1.

The elemental composition of fuel has a correlation with its properties. In particular, an increase in the oxygen content of the fuel was observed, rising from 5.3 % to 9.7 %, a rise of 73 %. Conversely, the carbon content saw a decrease of 5 % from 81.22 to 58.45 m/m with GEP10 compared to GEP0. This could be attributed to the higher oxygen content and lower carbon content of PODE1 compared with gasoline. Thereby, the heat of combustion decreased from 41.51 to 39.83, as shown in Fig. 3. The alteration in the oxygen, carbon, and hydrogen content was consistent with the findings reported in references. [29,30]. Also, it is noted from the data that the percentage of change with oxygen was much higher when the PODE1 additive increased compared to GEP0 (gasoline-ethanol) fuel, as shown in Fig. 4.

In summary, the study sheds light on key properties and implications of blending PODE1 with gasoline-ethanol (E10), showcasing potential benefits and effects on engine performance and emissions.

## 5 Conclusions

The present study aimed to examine the characteristics and stability of the PODE1 additive upon its incorporation into gasoline-ethanol (E10) blend fuels. The objective of this study was to evaluate the efficacy of PODE1 as an alternative option for reducing soot emissions in Gasoline Direct Injection (GDI) engines using E10 fuel blends.

The findings of this investigation revealed a noticeable pattern: with an increase in the ratio of PODE1 in the blend, significant indices such as Research Octane Number (RON), kinematic viscosity, carbon and hydrogen content, and heat of combustion exhibited a drop. It is worth mentioning that the E10 and PODE1 blends exhibited remarkable stability throughout the duration of the investigation, as shown by the absence of any indications of phase separation, even when the PODE1 concentrations reached as high as 10 %.In addition, the use of PODE1 at a concentration of up to 10 % had negligible effects on key characteristics of E10, such as its boiling point and octane number. The E10 and PODE1 blend significantly mitigated the potential disruption of engine performance caused by low boiling points, namely the occurrence of gasoline boiling in pumps and lines under high-temperature situations.

The use of PODE1 at levels of up to 10 % yielded significant reductions in emissions, particularly in relation to the release of soot from gasoline direct injection (GDI) engines. The aforementioned finding underlines the potential of PODE1 as an environmentally friendly addition in gasoline-ethanol blends, offering a feasible approach to mitigate the release of soot from GDI engines.This research presents PODE1 as a potential solution for improving gasoline-ethanol blends in order to reduce soot emissions from GDI engines. The next study phase will explore the complex effects of the environmentally beneficial PODE1 additive on both soot emissions and the overall performance of gasoline direct injection (GDI) engines. This research will provide more knowledge on cleaner and more efficient combustion technologies.

## CRediT authorship contribution statement

**Omar I. Awad:** Conceptualization, Data curation, Formal analysis. **Bo Zhou:** Investigation, Methodology. **Zhenbin Chen:** Funding acquisition, Supervision. **Kumaran Kadirgama:** Resources. **M.N. Mohammed:** Conceptualization, Writing – review & editing. **D. Ramasamy:** Funding acquisition.

## Declaration of competing interest

The authors confirm that there are no known conflicts of interest associated with this publication.
