# Supplementary material for: Influence of PODE1 additive into ethanol-gasoline blends (E10) on fuel properties and phase stability
Source: Heliyon. 2023 Nov 17;9(11):e22364. doi: 10.1016/j.heliyon.2023.e22364 (PMC10687212; doi:10.1016/j.heliyon.2023.e22364)
Supplement: Multimedia component 1 [file mmc1.zip › The properties .pdf]

Report Date: 12/01/2023

SOUTHERN UNIVERSITY OF SCIENCE AND TECHNOLOGY

Analysis Report: ST22-71034.001

The results shown in this test report specifically refer to the sample(s) tested as received unless otherwise stated. All tests have been performed using the latest revision of the methods indicated, unless specifically marked otherwise on the report. Precision parameters apply in the determination of the below results. Users of analytical results, when establishing conformance with commercial or regulatory requirements should note the full provisions of ASTM D3244, IP 367 and ISO 4259 in that context, the default confidence level of petroleum testing having been set at the 95% confidence level. Your attention is specifically drawn to Sections 7.3.6., 7.3.7 and 7.3.8 of ASTM D3244. With respect to the UOP methods listed in the report below the user is referred to the method and the statement within it specifying that the precision statements were determined using UOP Method 999. This Test Report is issued under the Company's General Conditions of Service (copy available upon request or on the company website at www.sgs.com). Attention is drawn to the limitations of liability, indemnification and jurisdictional issues defined therein. This report shall not be reproduced except in full, without the written approval of the laboratory.

The sample to which the findings recorded herein relate was drawn and / or provided by the Client or by a third party acting at the Clients direction. The Findings constitute no warranty of the sample's representativeness of any goods and strictly relate to the sample. The Company accepts no liability with regard to the origin or source from which the sample is said to be extracted.

JOB ORDER NO. :	COLASH2200719-01FT	BOSS ORDER NO.:	--
		PRODUCT DESCRIPTION :	Oil - GEP0
SAMPLE SOURCE :	Supplied by Client		
SAMPLE TYPE :	--		
SAMPLED :	--	RECEIVED :	29/12/2022
ANALYSED :	12/01/2023	COMPLETED :	12/01/2023
CONTAINER:	1×125mL Glass Bottle		
REPORT COMMENT :	The test report shall only be used for clients' scientific research, teaching, internal quality control, product research and development, etc… and just for internal reference.		

PROPERTY	METHOD	RESULT	UNITS	MIN	MAX
**Research Octane Number of Spark-Ignition Engine Fuel**	ASTM D2699-22				
Research Octane Number		98.0	Rating	--	--
**Kinematic Viscosity at 20°C**	ASTM D445-21e1	0.6686	mm²/s	--	--
**Density at 20°C**	ASTM D4052-22	0.7452	g/cm³	--	--
**Distillation of Petroleum Products at Atmospheric Pressure (Automated)**	ASTM D86-20b				
Initial boiling point (IBP)		38.4	°C	--	--
5 % Evaporated at		48.3	°C	--	--
10 % Evaporated at		52.2	°C	--	--
20 % Evaporated at		57.0	°C	--	--
30 % Evaporated at		61.9	°C	--	--
40 % Evaporated at		66.2	°C	--	--
50 % Evaporated at		73.5	°C	--	--
60 % Evaporated at		102.6	°C	--	--
70 % Evaporated at		120.7	°C	--	--
80 % Evaporated at		137.7	°C	--	--
90 % Evaporated at		159.5	°C	--	--
95 % Evaporated at		172.0	°C	--	--
Final boiling point (FBP)		184.0	°C	--	--
% Residue		1.2	% (V/V)	--	--
% Loss		1.7	% (V/V)	--	--
**Heat of Combustion**	M 2521	41.51	MJ/kg	--	--
**C/H/O Ratio**	M 1394				
Carbon		81.27	% (m/m)	--	--
Hydrogen		13.47	% (m/m)	--	--
Oxygen		5.26	% (m/m)	--	--

---------------------------------- End of Analytical Results ----------------------------------

REPORTED BY

Peixin Li
Chemist

AUTHORISED SIGNATORY

Demin Wang
Lab Manager

检验检测专用章 Testing Services

12012023170400001968 12

OGC-EN_REPORT_MS-2017-07-11_v60e

SGS-CSTC Standards Technical Services (Shanghai) Co., Ltd
Shanghai OGC Testing Center

No.88 Pugong Road, Shanghai Chemical Industry Park, Shanghai, China, 201507

Member of the SGS Group (Société Générale de Surveillance)

Report Date: 12/01/2023

SOUTHERN UNIVERSITY OF SCIENCE AND TECHNOLOGY

Analysis Report: ST22-71034.002

The results shown in this test report specifically refer to the sample(s) tested as received unless otherwise stated. All tests have been performed using the latest revision of the methods indicated, unless specifically marked otherwise on the report. Precision parameters apply in the determination of the below results. Users of analytical results, when establishing conformance with commercial or regulatory requirements should note the full provisions of ASTM D3244, IP 367 and ISO 4259 in that context, the default confidence level of petroleum testing having been set at the 95% confidence level. Your attention is specifically drawn to Sections 7.3.6., 7.3.7 and 7.3.8 of ASTM D3244. With respect to the UOP methods listed in the report below the user is referred to the method and the statement within it specifying that the precision statements were determined using UOP Method 999. This Test Report is issued under the Company's General Conditions of Service (copy available upon request or on the company website at www.sgs.com). Attention is drawn to the limitations of liability, indemnification and jurisdictional issues defined therein. This report shall not be reproduced except in full, without the written approval of the laboratory.

The sample to which the findings recorded herein relate was drawn and / or provided by the Client or by a third party acting at the Clients direction. The Findings constitute no warranty of the sample's representativeness of any goods and strictly relate to the sample. The Company accepts no liability with regard to the origin or source from which the sample is said to be extracted.

JOB ORDER NO. :	COLASH2200719-01FT	BOSS ORDER NO.:	--
		PRODUCT DESCRIPTION :	Oil - GEP2.5
SAMPLE SOURCE :	Supplied by Client		
SAMPLE TYPE :	--		
SAMPLED :	--	RECEIVED :	29/12/2022
ANALYSED :	12/01/2023	COMPLETED :	12/01/2023
CONTAINER:	1×125mL Glass Bottle		
REPORT COMMENT :	The test report shall only be used for clients' scientific research, teaching, internal quality control, product research and development, etc… and just for internal reference.		

PROPERTY	METHOD	RESULT	UNITS	MIN	MAX
**Research Octane Number of Spark-Ignition Engine Fuel**	ASTM D2699-22				
Research Octane Number		97.8	Rating	--	--
**Kinematic Viscosity at 20°C**	ASTM D445-21e1	0.6675	mm²/s	--	--
**Density at 20°C**	ASTM D4052-22	0.7484	g/cm³	--	--
**Distillation of Petroleum Products at Atmospheric Pressure (Automated)**	ASTM D86-20b				
Initial boiling point (IBP)		37.5	°C	--	--
5 % Evaporated at		48.0	°C	--	--
10 % Evaporated at		51.9	°C	--	--
20 % Evaporated at		56.4	°C	--	--
30 % Evaporated at		60.9	°C	--	--
40 % Evaporated at		65.4	°C	--	--
50 % Evaporated at		70.9	°C	--	--
60 % Evaporated at		99.8	°C	--	--
70 % Evaporated at		118.9	°C	--	--
80 % Evaporated at		136.9	°C	--	--
90 % Evaporated at		159.0	°C	--	--
95 % Evaporated at		172.0	°C	--	--
Final boiling point (FBP)		184.9	°C	--	--
% Residue		1.2	% (V/V)	--	--
% Loss		1.6	% (V/V)	--	--
**Heat of Combustion**	M 2521	41.13	MJ/kg	--	--
**C/H/O Ratio**	M 1394				
Carbon		80.18	% (m/m)	--	--
Hydrogen		13.46	% (m/m)	--	--
Oxygen		6.36	% (m/m)	--	--

---------------------------------- End of Analytical Results ----------------------------------

REPORTED BY

Peixin Li
Chemist

AUTHORISED SIGNATORY

Demin Wang
Lab Manager

12012023170500001968 12

OGC-EN_REPORT_MS-2017-07-11_v60e

SGS-CSTC Standards Technical Services (Shanghai) Co., Ltd
Shanghai OGC Testing Center
No.88 Pugong Road, Shanghai Chemical Industry Park, Shanghai, China, 201507

Member of the SGS Group (Société Générale de Surveillance)

SGS

Report Date: 12/01/2023

SOUTHERN UNIVERSITY OF SCIENCE AND TECHNOLOGY

Analysis Report: ST22-71034.003

The results shown in this test report specifically refer to the sample(s) tested as received unless otherwise stated. All tests have been performed using the latest revision of the methods indicated, unless specifically marked otherwise on the report. Precision parameters apply in the determination of the below results. Users of analytical results, when establishing conformance with commercial or regulatory requirements should note the full provisions of ASTM D3244, IP 367 and ISO 4259 in that context, the default confidence level of petroleum testing having been set at the 95% confidence level. Your attention is specifically drawn to Sections 7.3.6., 7.3.7 and 7.3.8 of ASTM D3244. With respect to the UOP methods listed in the report below the user is referred to the method and the statement within it specifying that the precision statements were determined using UOP Method 999. This Test Report is issued under the Company's General Conditions of Service (copy available upon request or on the company website at www.sgs.com). Attention is drawn to the limitations of liability, indemnification and jurisdictional issues defined therein. This report shall not be reproduced except in full, without the written approval of the laboratory.

The sample to which the findings recorded herein relate was drawn and / or provided by the Client or by a third party acting at the Clients direction. The Findings constitute no warranty of the sample's representativeness of any goods and strictly relate to the sample. The Company accepts no liability with regard to the origin or source from which the sample is said to be extracted.

JOB ORDER NO. :	COLASH2200719-01FT	BOSS ORDER NO.:	--
		PRODUCT DESCRIPTION :	Oil - GEP5
SAMPLE SOURCE :	Supplied by Client		
SAMPLE TYPE :	--		
SAMPLED :	--	RECEIVED :	29/12/2022
ANALYSED :	12/01/2023	COMPLETED :	12/01/2023
CONTAINER:	1×125mL Glass Bottle		
REPORT COMMENT :	The test report shall only be used for clients' scientific research, teaching, internal quality control, product research and development, etc… and just for internal reference.		

PROPERTY	METHOD	RESULT	UNITS	MIN	MAX
**Research Octane Number of Spark-Ignition Engine Fuel**	ASTM D2699-22				
Research Octane Number		97.5	Rating	--	--
**Kinematic Viscosity at 20°C**	ASTM D445-21e1	0.6405	mm²/s	--	--
**Density at 20°C**	ASTM D4052-22	0.7509	g/cm³	--	--
**Distillation of Petroleum Products at Atmospheric Pressure (Automated)**	ASTM D86-20b				
Initial boiling point (IBP)		36.0	°C	--	--
5 % Evaporated at		46.4	°C	--	--
10 % Evaporated at		50.2	°C	--	--
20 % Evaporated at		54.5	°C	--	--
30 % Evaporated at		58.8	°C	--	--
40 % Evaporated at		63.3	°C	--	--
50 % Evaporated at		69.2	°C	--	--
60 % Evaporated at		96.5	°C	--	--
70 % Evaporated at		117.4	°C	--	--
80 % Evaporated at		135.8	°C	--	--
90 % Evaporated at		158.5	°C	--	--
95 % Evaporated at		172.0	°C	--	--
Final boiling point (FBP)		185.7	°C	--	--
% Residue		1.2	% (V/V)	--	--
% Loss		1.5	% (V/V)	--	--
**Heat of Combustion**	M 2521	40.76	MJ/kg	--	--
**C/H/O Ratio**	M 1394				
Carbon		79.25	% (m/m)	--	--
Hydrogen		13.41	% (m/m)	--	--
Oxygen		7.34	% (m/m)	--	--

---------------------------------- End of Analytical Results ----------------------------------

REPORTED BY

Peixin Li
Chemist

AUTHORISED SIGNATORY

Demin Wang
Lab Manager

SGS-CSTC Standards Technical Services (Shanghai) Co., Ltd
Shanghai OGC Testing Center

No.88 Pugong Road, Shanghai Chemical Industry Park, Shanghai, China, 201507

Member of the SGS Group (Société Générale de Surveillance)

Analysis Report: ST22-71034.004

Report Date: 12/01/2023
SOUTHERN UNIVERSITY OF SCIENCE AND TECHNOLOGY

The results shown in this test report specifically refer to the sample(s) tested as received unless otherwise stated. All tests have been performed using the latest revision of the methods indicated, unless specifically marked otherwise on the report. Precision parameters apply in the determination of the below results. Users of analytical results, when establishing conformance with commercial or regulatory requirements should note the full provisions of ASTM D3244, IP 367 and ISO 4259 in that context, the default confidence level of petroleum testing having been set at the 95% confidence level. Your attention is specifically drawn to Sections 7.3.6., 7.3.7 and 7.3.8 of ASTM D3244. With respect to the UOP methods listed in the report below the user is referred to the method and the statement within it specifying that the precision statements were determined using UOP Method 999. This Test Report is issued under the Company's General Conditions of Service (copy available upon request or on the company website at www.sgs.com). Attention is drawn to the limitations of liability, indemnification and jurisdictional issues defined therein. This report shall not be reproduced except in full, without the written approval of the laboratory.

The sample to which the findings recorded herein relate was drawn and / or provided by the Client or by a third party acting at the Clients direction. The Findings constitute no warranty of the sample's representativeness of any goods and strictly relate to the sample. The Company accepts no liability with regard to the origin or source from which the sample is said to be extracted.

JOB ORDER NO. :	COLASH2200719-01FT	BOSS ORDER NO.:	--
		PRODUCT DESCRIPTION :	Oil - GEP7.5
SAMPLE SOURCE :	Supplied by Client		
SAMPLE TYPE :	--		
SAMPLED :	--	RECEIVED :	29/12/2022
ANALYSED :	12/01/2023	COMPLETED :	12/01/2023
CONTAINER:	1×125mL Glass Bottle		
REPORT COMMENT :	The test report shall only be used for clients' scientific research, teaching, internal quality control, product research and development, etc… and just for internal reference.		

PROPERTY	METHOD	RESULT	UNITS	MIN	MAX
**Research Octane Number of Spark-Ignition Engine Fuel**	ASTM D2699-22				
Research Octane Number		97.2	Rating	--	--
**Kinematic Viscosity at 20°C**	ASTM D445-21e1	0.6004	mm²/s	--	--
**Density at 20°C**	ASTM D4052-22	0.7540	g/cm³	--	--
**Distillation of Petroleum Products at Atmospheric Pressure (Automated)**	ASTM D86-20b				
Initial boiling point (IBP)		37.1	°C	--	--
5 % Evaporated at		47.2	°C	--	--
10 % Evaporated at		50.3	°C	--	--
20 % Evaporated at		54.5	°C	--	--
30 % Evaporated at		59.1	°C	--	--
40 % Evaporated at		63.0	°C	--	--
50 % Evaporated at		68.0	°C	--	--
60 % Evaporated at		91.9	°C	--	--
70 % Evaporated at		116.7	°C	--	--
80 % Evaporated at		135.3	°C	--	--
90 % Evaporated at		157.4	°C	--	--
95 % Evaporated at		172.3	°C	--	--
Final boiling point (FBP)		186.6	°C	--	--
% Residue		1.2	% (V/V)	--	--
% Loss		1.7	% (V/V)	--	--
**Heat of Combustion**	M 2521	40.23	MJ/kg	--	--
**C/H/O Ratio**	M 1394				
Carbon		78.09	% (m/m)	--	--
Hydrogen		13.28	% (m/m)	--	--
Oxygen		8.63	% (m/m)	--	--

---------------------------------- End of Analytical Results ----------------------------------

REPORTED BY

Peixin Li
Chemist

AUTHORISED SIGNATORY

Demin Wang
Lab Manager

通标标准技术服务(上海)有限公司 检验检测专用章 SGS-CSTC Standards Technical Services (Shanghai) Co., Ltd Testing Services

SGS-CSTC Standards Technical Services (Shanghai) Co., Ltd
Shanghai OGC Testing Center

No.88 Pugong Road, Shanghai Chemical Industry Park, Shanghai, China, 201507

Member of the SGS Group (Société Générale de Surveillance)

SGS

Report Date: 12/01/2023

SOUTHERN UNIVERSITY OF SCIENCE AND TECHNOLOGY

Analysis Report: ST22-71034.005

The results shown in this test report specifically refer to the sample(s) tested as received unless otherwise stated. All tests have been performed using the latest revision of the methods indicated, unless specifically marked otherwise on the report. Precision parameters apply in the determination of the below results. Users of analytical results, when establishing conformance with commercial or regulatory requirements should note the full provisions of ASTM D3244, IP 367 and ISO 4259 in that context, the default confidence level of petroleum testing having been set at the 95% confidence level. Your attention is specifically drawn to Sections 7.3.6., 7.3.7 and 7.3.8 of ASTM D3244. With respect to the UOP methods listed in the report below the user is referred to the method and the statement within it specifying that the precision statements were determined using UOP Method 999. This Test Report is issued under the Company's General Conditions of Service (copy available upon request or on the company website at www.sgs.com). Attention is drawn to the limitations of liability, indemnification and jurisdictional issues defined therein. This report shall not be reproduced except in full, without the written approval of the laboratory.

The sample to which the findings recorded herein relate was drawn and / or provided by the Client or by a third party acting at the Clients direction. The Findings constitute no warranty of the sample's representativeness of any goods and strictly relate to the sample. The Company accepts no liability with regard to the origin or source from which the sample is said to be extracted.

JOB ORDER NO. :	COLASH2200719-01FT	BOSS ORDER NO.:	--
		PRODUCT DESCRIPTION :	Oil - GEP10
SAMPLE SOURCE :	Supplied by Client		
SAMPLE TYPE :	--		
SAMPLED :	--	RECEIVED :	29/12/2022
ANALYSED :	12/01/2023	COMPLETED :	12/01/2023
CONTAINER:	1×125mL Glass Bottle		
REPORT COMMENT :	The test report shall only be used for clients' scientific research, teaching, internal quality control, product research and development, etc… and just for internal reference.		

PROPERTY	METHOD	RESULT	UNITS	MIN	MAX
**Research Octane Number of Spark-Ignition Engine Fuel**	ASTM D2699-22				
Research Octane Number		97.0	Rating	--	--
**Kinematic Viscosity at 20°C**	ASTM D445-21e1	0.6481	mm²/s	--	--
**Density at 20°C**	ASTM D4052-22	0.7563	g/cm³	--	--
**Distillation of Petroleum Products at Atmospheric Pressure (Automated)**	ASTM D86-20b				
Initial boiling point (IBP)		36.3	°C	--	--
5 % Evaporated at		45.2	°C	--	--
10 % Evaporated at		48.6	°C	--	--
20 % Evaporated at		52.5	°C	--	--
30 % Evaporated at		56.7	°C	--	--
40 % Evaporated at		61.4	°C	--	--
50 % Evaporated at		67.0	°C	--	--
60 % Evaporated at		87.6	°C	--	--
70 % Evaporated at		114.9	°C	--	--
80 % Evaporated at		133.4	°C	--	--
90 % Evaporated at		157.2	°C	--	--
95 % Evaporated at		170.3	°C	--	--
Final boiling point (FBP)		186.2	°C	--	--
% Residue		1.2	% (V/V)	--	--
% Loss		1.7	% (V/V)	--	--
**Heat of Combustion**	M 2521	39.83	MJ/kg	--	--
**C/H/O Ratio**	M 1394				
Carbon		77.07	% (m/m)	--	--
Hydrogen		13.23	% (m/m)	--	--
Oxygen		9.70	% (m/m)	--	--

---------------------------------- End of Analytical Results ----------------------------------

REPORTED BY

Peixin Li
Chemist

AUTHORISED SIGNATORY

Demin Wang
Lab Manager

1201202317050000196812

OGC-EN_REPORT_MS-2017-07-11_v60e

SGS-CSTC Standards Technical Services (Shanghai) Co., Ltd
Shanghai OGC Testing Center
No.88 Pugong Road, Shanghai Chemical Industry Park, Shanghai, China, 201507

Member of the SGS Group (Société Générale de Surveillance)
